# Overcoming arguments against tackling child poverty and inequalities: marshalling the evidence

**DOI:** 10.1136/bmjpo-2026-004714

**Published:** 2026-11-02

**Authors:** Maximilian Limburg, Mary Cronin, Kate E Pickett, David Taylor-Robinson

**Affiliations:** 1 Department of Public Health, Policy and Systems, University of Liverpool, Liverpool, UK; 2 Department of Health Sciences, University of York, York, UK

**Keywords:** Child Health, Health Policy, Adolescent Health, Child

## Abstract

Child poverty is a significant and growing issue in the UK, with nearly one in three children now living in poverty. The detrimental effects on children’s physical and mental health, as well as the perpetuation of wider inequalities, are well documented. However, instead of renewed efforts to alleviate this, policymakers and advocates continue to face staunch opposition to comprehensively tackling child poverty, often rooted in longstanding misconceptions, reinforced by the media and political discourse. To overcome this resistance, this article marshals the evidential basis for three structural truths: that social investment is a critical driver of economic growth; that structural environment, rather than individual failings, dictates family outcomes; and that domestic policy and investment, not demographics, define the availability of housing and public services. By anchoring our analysis in these evidence-based realities, we address and dismantle the dominant narratives of economic scarcity, parental blame and deflection that frequently stall effective reform. Ultimately, equipping policymakers and advocates with this evidence base strengthens the case for sustained, structural action to reduce child poverty and promote child health equity in the UK.

**WHAT IS KNOWN ABOUT THIS TOPIC**

- Rising child poverty in the UK now affects nearly one in three children, severely harming their health, education and long-term life chances.
- Policy efforts to alleviate this crisis frequently encounter resistance rooted in persistent misconceptions, leading to detrimental inaction that pushes more children into poverty.

**WHAT THIS STUDY ADDS**

- Our review marshals evidence to establish three realities: social investment drives economic growth, structural inequalities constrain family choices and domestic policy controls public service capacity.
- We critically assess and dismantle narratives opposing poverty action, specifically arguments of economic scarcity, parental blame and the deflection of systemic failure onto external factors.
- This article provides an evidence-based resource for policymakers and advocates to counter these common misconceptions, strengthening the case for sustained action promoting child health equity.

## Introduction

 In the UK government’s first review of health inequalities, the Black Report of 1980, the first policy recommendation was to ‘give children a better start in life’, a call that has been re-iterated in the 45 years since. However, in the last 15 years, the number of children living in poverty has increased by 900 000 to 4.5 million, to almost one in three children.^1^ This trend is illustrated in figure 1, which shows that the percentage of children in relative poverty has risen to 31%, surpassing all other age groups. Rather than actively tackling this growing issue, the amount of money invested in welfare, early years and education for children has decreased in real terms between 2010 and 2021.^2 3^ The only significant reduction in poverty during this period was driven by the marked increase in income and social protections during the 2020–2021 pandemic years.^4^

**Figure 1:**
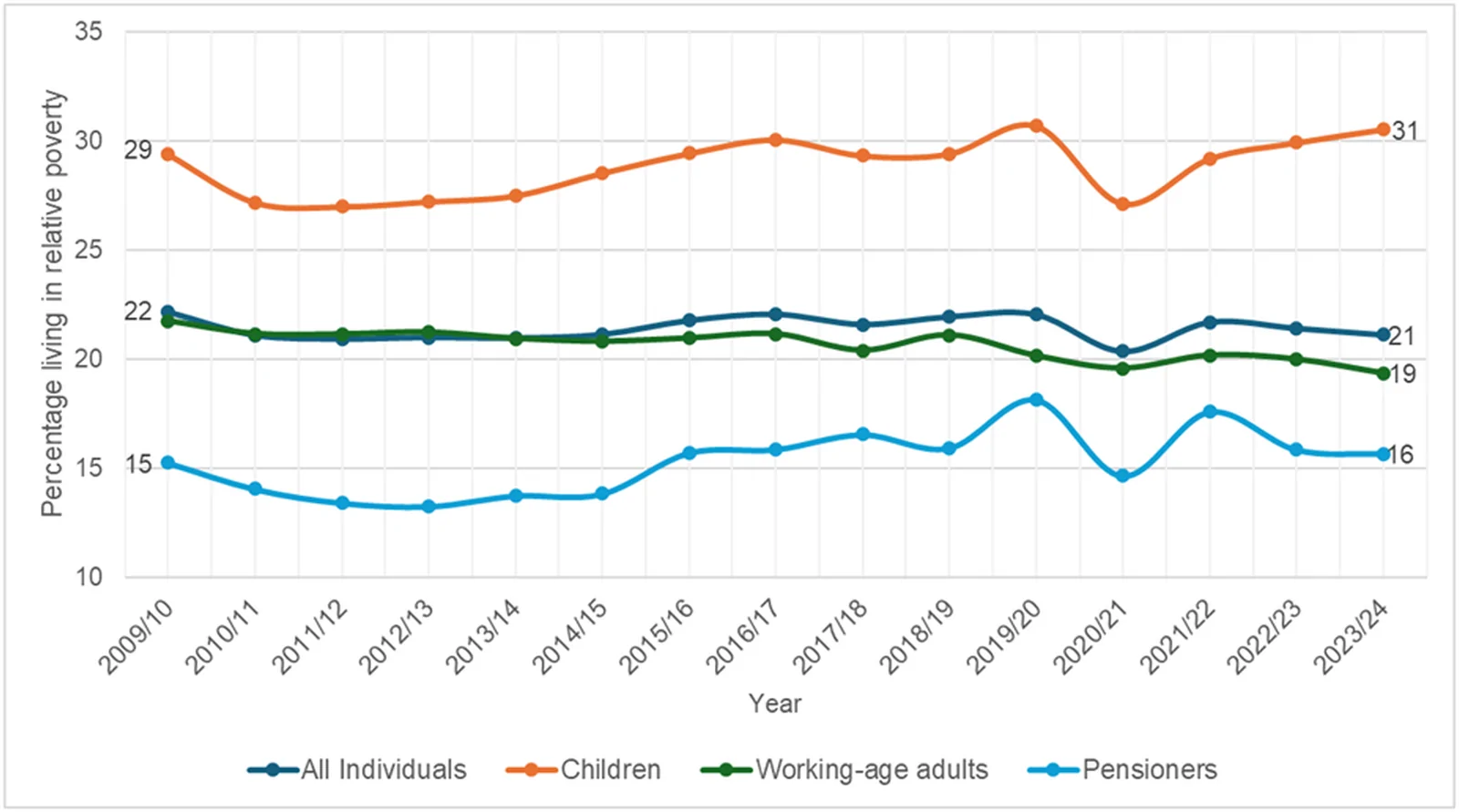
Percentage of individuals living in households in relative poverty (after housing costs).^75^

This lack of investment has detrimental consequences for children growing up in the UK and wider society. A child born in the poorest areas of England has a life expectancy almost 10 years less and a healthy life expectancy almost 20 years less than a child born in the richest, with these gaps further increasing between 2015 and 2020.^5^ There is a clear social gradient in the health, educational attainment and life chances of children. An analysis of the UK Millennium Cohort Study showed that children experiencing persistent poverty, compared with those in low poverty and adversity, have twice the odds of cognitive disability, obesity or drug experimentation by age 14 and threefold the chance of socioemotional behavioural problems.^6^ The economic burden of child poverty in the UK is substantial, estimated to reach £40 billion annually by 2027, even in conservative estimates. This cost is made up of £13 billion in future lost earnings, a cost of £5 billion to the treasury in lost income tax, £2 billion in unemployment benefits and £17 billion in universal, as well as £3 billion in poverty focused public spending.^7^

Despite extensive evidence of the adverse consequences, tackling child poverty remains a polarising political debate, with the absence of a unified consensus standing in the way of effective long-term measures to alleviate this strain on children. Across successive political cycles, a persistently high level of child poverty appears to have become implicitly accepted, with government policies largely restricted to marginal adjustments. The 14 years of Coalition and Conservative governments between 2010 and 2024 saw the removal of targets to reduce financial poverty in children in 2016,^8 9^ the introduction of the two-child benefit cap in 2017 and the attempted reframing of health inequalities as disparities.^10^ The reform of the tax and welfare system, alongside hundreds of smaller amendments to entitlement criteria altering the financial safety nets provided to struggling families, ultimately resulted in the poorest fifth of households experiencing a 10.2% reduction of income between 2010 and 2025.^11^ The 2024 Labour government initiated a renewed policy drive to create ‘the healthiest and happiest generation of children ever’.^12^ However, their ‘ambitious strategy to reduce child poverty’^13^ was not published until December 2025. This timeline, with implementation beginning in April 2026, meant that pre-existing poverty trajectories continued unabated for two additional winters. Recent estimates suggest that the strategy will reduce child poverty to 29% by the end of the parliamentary period in 2030.^14^ Nevertheless, the retention of the overall benefit cap and the freeze on local housing allowance constrain this impact, with children persistently remaining as the most disproportionately impoverished demographic group.^15^ This incremental reduction, solely altering poverty at the margins, leaves vulnerable households with a minimal buffer against possible macro-economic deterioration, such as through escalating inflation, geopolitical upheaval or immediate reversal through political change.

While robust and extensive evidence regarding the causes of child poverty and potential solutions exists, effective intervention continues to be frequently stalled by a persistent set of opposing narratives. Especially in an era of increasingly digital media and polarised politics, emotive stories that rely on concepts of scarcity, blame and deflection tend to dominate political discourse, capturing the public consciousness far more effectively than the complex structural truths underlying persistent poverty, creating a barrier to evidence-based reform.

To ensure this review addresses the actual barriers faced by policymakers and public health professionals, we identified three dominant narratives through a deliberative workshop held in October 2024. This workshop convened six participants, including academics from the University of Liverpool and University of York, alongside communications and public affairs staff from the Northern Health Science Alliance, who brought collective expertise in child health, health inequalities and both national and international policy. During a facilitated group discussion, participants reflected on their experiences with child poverty advocacy, political interactions and responses to proposed public health interventions. These reflections were refined via consensus to isolate the most dominant themes. This was further validated with a targeted review of UK media discourse from 2010 to 2025, identifying digitally published media through internet searches (online supplemental tables S1 and S2). While other well-described frames exist within UK policy discourse, these narratives were selected as they represented the most salient barriers encountered in practice by workshop advocates. We acknowledge that the identification of these specific narratives relies on a degree of author and participant judgement; however, it provides a targeted mechanism for this review rather than attempting an exhaustive analysis of all political framing.

Through this process we established that advocates for child equity frequently encounter three entrenched arguments that attempt to justify policy inaction. First, the argument of economic scarcity suggests that social investment, such as increased public spending on services like early years support or Universal Credit, is a luxury that must wait for sufficient economic growth or even acts as an inhibitor to competitiveness. Second, the narrative of blame frames structural deprivation as a failure of parental responsibility or lifestyle choices. Finally, the tactic of deflection erroneously attributes housing shortages and a strain on public services to external factors, such as immigration, rather than longstanding domestic policy decisions and underinvestment. By marshalling the evidential basis against these specific narratives, we argue that social investment is actually a driver of growth; that it is structural environments rather than individual failings that dictate family outcomes; and that domestic policy, not demographics, defines the housing market and availability of public services.

### Social investment as a driver of growth

There is robust evidence indicating that measures to directly address child poverty and inequalities deliver economic benefits at a fraction of the cost of inaction, both in terms of productivity and future economic output.^16^ Some measures yield immediate benefits, like abolishing the two-child limit on Universal Credit in April 2026, which is predicted to instantly lift 350 000 children out of poverty and prevent a further 150 000 children from falling into poverty prior to the end of the parliamentary term.^17^ The estimated £2 billion required to enact this policy change represents a fraction of the broader economic burden, constituting just 5% of the £40 billion annual cost attributed to child poverty.^7 17^ Direct action on regional inequalities can also have benefits for the economy. Increasing the National Health Service (NHS) budget by 10% in Northern England, which has an almost 2-year gap in life expectancy and consistently worse health outcomes compared with the rest of England, would decrease economic inactivity rates by 3% and improve median weekly pay by 1%. This action would directly lift children out of poverty, while eliminating the differential impact of poor health on employment in the region, adding £13.2 billion in gross value to the UK economy.^18^

Despite this, policy action is often stalled by concerns that social spending dampens economic competitiveness, due to increased taxes or borrowing required to fund a strong welfare system, with this stance narratively reinforced by the absence of the purported ‘magic money tree’. These arguments have persisted throughout the entire history of debating health inequalities, with concerns about cost implications and funding already present when the Black Report was published during the Thatcher administration in 1980, continuing to the 2025 child poverty strategy being delayed by the current Labour Administration, citing identical reasons.^19^

However, these narratives have been repeatedly disproven by the experience of other high-income nations. Nordic states provide comprehensive social protection through universal healthcare, education and generous unemployment benefits, significantly reducing poverty and inequality, while maintaining high levels of population health, social mobility and economic competitiveness.^20^ These systems create strong social cohesion, enabling higher tax acceptance in exchange for extensive public services, while also supporting family well-being through policies like generous parental leave and subsidised childcare that enable full workforce participation. Evidence suggests that these wide-reaching social policies cultivate a more productive workforce and stimulate innovative entrepreneurship, allowing citizens to thrive economically and acting as a catalyst for long-term growth.^21^ Maintaining these social measures during times of major crisis, even increasing policy coverage during the pandemic, does not appear to harm economic competitiveness either, with Nordic countries consistently outperforming European and Organisation for Economic Co-operation and Development (OECD) averages. When ranking Gross Domestic Product (GDP) per capita across European countries, Norway ranks 4th, Denmark 6th and Sweden 10th, compared with the UK at 14th with a GDP per capita of approximately US$35 000 less than Norway.^22^

Even when evaluated beyond comparisons to countries with the world’s strongest welfare systems, the UK consistently underperforms. Between 2012 and 2021, the UK experienced the largest increase in child poverty among all assessed OECD and EU countries, as visualised in figure 2, registering a rise 8.6 percentage points higher than the nation with the second-highest rise.^23^ While some comparable economies, like France, also experienced increases, although at a substantially lower rate, others, like Canada or Japan, managed to achieve meaningful progress, recording reductions of nearly 20% in the same period. Canada, in particular, which shares an almost identical Gross National Income (GNI) per capita with the UK, stands out for having achieved the exact inverse of the UK’s trajectory. This reduction was not driven by macroeconomic conditions alone, but by targeted structural reform of the welfare system, primarily the 2016 introduction of the Canada Child Benefit.^24^ This success demonstrates that reversing child poverty is fundamentally a matter of domestic policy choice rather than economic inevitability, echoing former UN Special Rapporteur Philip Alston’s suggestion that poverty in the UK ‘is a political choice’.^25^ Within the UK, Scotland provides a clear domestic counterexample. Scottish child poverty rates are significantly lower at 21% than the wider UK picture, driven by targeted interventions to reduce housing costs, as well as progressive tax and benefit changes, such as the Scottish Child Payment, further proving that domestic policy adjustments can yield immediate improvements.^26^ Furthermore, prioritising economic growth over building a sustainable welfare system overlooks the risks associated with continued inequitable distribution of wealth, which directly exacerbates inequalities in health outcomes and the social determinants of health. Moreover, pursuit of growth at any cost can result in detrimental growth, generating economic patterns that undermine working conditions, erode social cohesion^27^ and contribute to unhealthy lifestyles and environmental degradation.^28^

**Figure 2:**
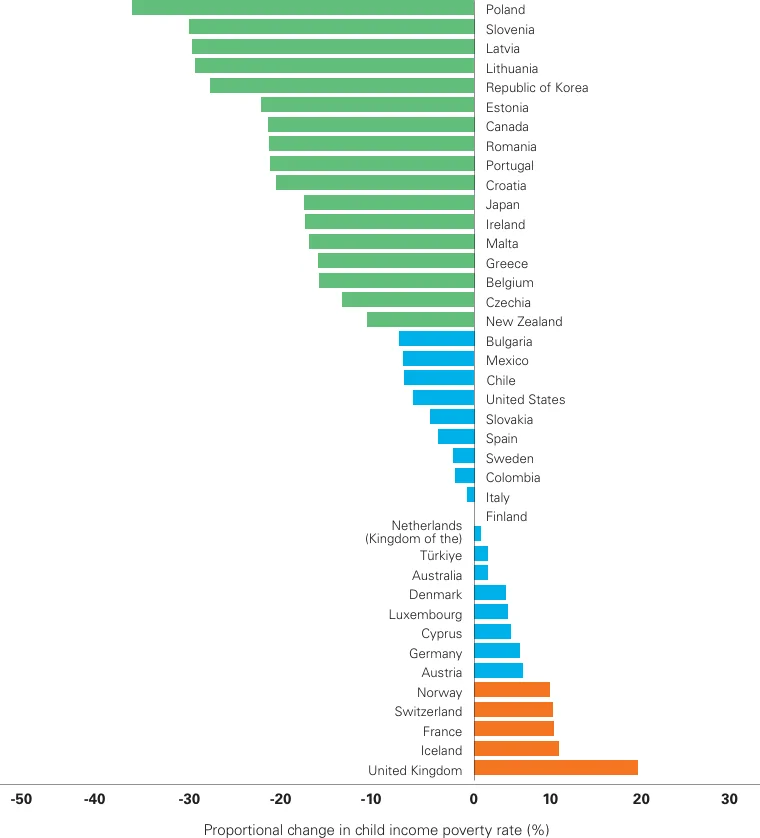
Proportional change in child poverty rates, 2012–2014 to 2019–2021.^23^

### Family well-being and structural inequalities

Evidence consistently demonstrates that being born into socioeconomic disadvantage is strongly associated with worse child well-being, quality of life, social cohesion, mental health and an increased risk of debt and insecure employment in adulthood.^29^ Poorer families face consistent structural inequalities that constrain choices and opportunities, with measurable consequences for children’s health. Research shows that households with children living in the poorest fifth of the population would need to spend 70% of their disposable income on food to meet the UK government’s healthy eating guidelines.^30^ Healthy foods are, on average, twice as expensive as unhealthy food, while fast-food outlets are more densely concentrated in deprived areas.^31^ Income also has a direct impact on housing choices. Families in poverty are more likely to live in socially or privately rented accommodation,^32^ often in areas with higher crime rates,^33^ limited access to greenspace^34^ and poor public transport links,^35^ directly impacting on a child’s ability to engage in physical activity, affecting mental health and educational attainment, while increasing rates of overweight and obesity.

In stark contrast to these structural realities, persistent narratives continue to rely on a deficit model, attributing poverty and its associated outcomes directly to parental irresponsibility and behavioural shortcomings. Within this framing, government interventions to support families in need are often criticised for supposedly cultivating dependency and rewarding a low work ethic across generations. Such arguments maintain that comprehensive welfare systems disrupt the mechanisms of meritocracy, where it is assumed that individual ability and determination are the primary drivers of social mobility.

Instead of generating dependency on the welfare state, evidence instead shows that in families with sufficient social support, children’s social mobility and later their adult income improves.^36^ Families in poverty continue to engage in the labour market with 65% of children in poverty having at least one working parent, while an estimated £22.7 billion in benefits across the UK went unclaimed altogether.^37^ This high proportion of in-work poverty highlights the distinction between a minimum wage and a real living wage. When statutory minimums fail to meet the actual cost of living, benefits paid to full-time employees effectively serve as a government subsidy for businesses paying the lowest wages possible. Furthermore, data on welfare recipients directly contradict the misconception that parents receiving benefits misuse their funds.^38^ Reviews of cash transfer policies worldwide have shown that recipients spent no more or significantly less money on alcohol and tobacco, instead choosing spending on more nutritious food and children’s goods.^39^ Analysis of family spending in the UK from 2023 shows that those in the bottom quintile of earners, a group with a high degree of overlap with benefit recipients, primarily spend their money on housing, fuel, food, transport and non-alcoholic beverages.^40^ Meanwhile an analysis of additional cost-of-living support payments received by benefit earners in 2024 showed they were primarily spent on utility bills and food or groceries.^41^ Where no cash transfer is involved, parents still appear to prioritise supporting children. A recent survey revealed that nearly half of UK parents are skipping meals to ensure their children can eat.^42^ This trend is particularly pronounced among young parents aged 16–24, with 73% reporting they have gone without food for their children’s sake.

Meritocratic narratives portray education as the universal pathway to social mobility, implying that free access creates a level playing field for children.^43^ In reality, opportunities to benefit from education are far from equally distributed.^44^ Schools in more affluent areas are three times more likely to be rated ‘outstanding’ compared with those in the most deprived communities, particularly at secondary level.^45^ The Programme for International Student Assessment (PISA) study, which tests maths, reading and science at age 15, showed that in English schools there was a more than 80 (out of 850) exam point difference between those in top and bottom socioeconomic quintiles across all three subjects.^46^ These inequalities persist to tertiary education, with a 43% gap in university attendance between most and least socioeconomically advantaged quintiles in England.^47^ Achieving a university degree has the potential to perpetuate the cycle of privilege, with children of parents with a degree twice as likely to be taking two or more A-level subjects preferred by universities compared with other students.^47^ Addressing these educational inequalities requires structural interventions that tackle broad social and economic determinants shaping children’s learning environments, ensuring that every child can reach their full potential.

### Structural capacity in housing and public services

Worsening affordability pressures and shortages on the housing market are driven by a complex mixture of domestic factors. The government’s analysis of house prices between 1999 and 2016 rates the primary drivers as income growth, interest rates and population growth.^48^ In addition to this, fewer social homes are being built, with the total number of households in social housing in 2024 having fallen by 1.4 million since 1980, further depleted by the Right to Buy scheme.^49^ In 2024, the government only invested £11.5 billion in its affordable homes programme, underachieving on the 90 000 new properties required to keep up with demand, while conversely spending £58.2 billion on housing support for private landlords.^50^

Despite limited empirical support, current political and media discourse often obscure domestic policy failures by attributing the strain on housing, employment opportunities and public services to external factors such as immigration. While net migration has been positive every year since 1994,^51 52^ it has risen sharply in recent years, driven by geopolitical instability, postpandemic economic shifts and humanitarian needs. By December 2024, it reached more than four times the levels seen prepandemic.^52^ However, narratives focus disproportionately on irregular migration, conflating 1 million legal immigrants with the approximately 45 000 irregular arrivals recorded in 2024.^52 53^ This misrepresentation has fuelled public hostility towards migrants, culminating in outbreaks of anti-immigration unrest across England in August 2024.

Speculative property markets and lack of regulation have given power to landlords, allowing rents to increase faster than incomes, with private rents increasing twice as fast as monthly total pay between January 2021 and 2025.^54 55^ However, this cost increase does not appear driven by migration, with research finding that local housing costs decrease in areas with higher immigration,^56^ while an increase in asylum seekers also reduces prices.^57^ Moreover, most migrants are ineligible for social housing on arrival and typically use tied accommodation linked to employers or houses of multiple occupancy via the private rented market, while asylum seekers are not eligible for council housing until after formal acceptance as refugees.^58^ Furthermore, there is no evidence that immigration has a harmful effect on the native workforce. This argument was already disproven pre-Brexit, when research found that areas with high EU migration saw no significant fall in employment or wages for UK-born workers.^59^ Evidence shows that migrants are up to twice as likely to start businesses compared with UK-born individuals, helping to create jobs.^60^ Additionally, most research shows that migrants contribute more to taxes and the economy than they consume in public services, while pessimistic estimates find a negligible negative effect of less than 1% on GDP.^61^

Media narratives often focus on the cost associated with rising immigration. The overall budget for the Home Office, who manage asylum, immigration, visas and passports, was £9.3 billion in 2023–2024, of which £3 billion was spent on housing asylum seekers in hotels while awaiting decisions on their claims.^62^ This represents 0.5% of government spending, while child poverty costs the UK £39.5 billion annually.^63^ Additionally, the number of hotel-accommodated asylum seekers is reducing, from a peak of 56 000 to 32 345 by March 2025.^64^ This disproportionate focus on costs often occurs during periods of austerity, deflecting blame for systemic failures onto migrants. British history shows a pattern of refugees, regardless of their ethnic origin, becoming scapegoats during times of national crises, such as in the 1890s, after World War II and in the 1990s/2000s.^65^ Public resentment is stoked by the perception that migrants receive special provisions, while the local population endures cuts to public services, exemplified by protests around asylum seeker hotels. Such protests ignore the nature of these accommodations, often more akin to army barracks than the hotels before conversion.^66^ Crucially, this narrative obscures the underlying causes, such as asylum processing backlogs and the disproportionate placement of migrants in already deprived areas.^67^ Blaming immigration, whether legal or irregular, for pressures on the welfare state, distracts from addressing inequitable wealth distribution and perpetuates the inequalities that sustain child poverty in the UK.

## Conclusions

Child poverty in the UK is a severe and escalating crisis, now affecting almost one in three children, demanding immediate and effective evidence-based structural intervention. Both the consequences of children continuing to live in poverty and the structural challenges perpetuating this problem are evident when examining the available data, as is the conclusion that the lack of progress has been primarily a political choice for large parts of the last two decades.^25^ Despite counternarratives of parental blame and attempted deflection onto external factors, it is clear that structural inequalities and long-standing domestic policy decisions are the primary drivers of continued poverty. Meanwhile, social investment offers an opportunity as a critical catalyst for economic growth, rather than inhibiting it. By firmly anchoring policy design in these outlined structural truths, advocates and policymakers can systematically dismantle the unfounded narratives of scarcity, blame and deflection that continue to stall meaningful progress.

Nevertheless, these misconceptions have persisted for decades and are likely to stay, while their reach and influence is further amplified by the evolving digital media landscape. Research has highlighted how sentiments across social and traditional mass media mutually reinforce one another, collectively shaping public emotion and opinion.^68^ Politicians themselves are beginning to use digital media to shape public sentiment and exert pressure on fellow policymakers.^69^ The recent COVID-19 pandemic highlighted the dangers and impact that misinformation, whether purposeful or accidental, can have.^70^

In this context, national and local policymakers will continue to confront public belief in misconceptions already refuted by evidence. Such narratives often function to suppress constructive debate on practical solutions before specific proposals can gain traction. However, continued inaction is detrimental, directly harming children and complicating future policy by raising costs. Where actions are taken, they are frequently delayed or diminished in impact, further exacerbating poverty for vulnerable families. For example, before the abolition of the two-child benefit cap, a measure available at a fraction of annual child poverty costs in the UK, there were 21 months of inaction between the election and implementation, which saw additional families pushed into poverty. Although a flagship policy that is due to have significant impact once fully implemented, it remains questionable whether this intervention alone is enough to reverse a decade of austerity and sustainably build towards an equitable future.

While the full benefits of some policies may not be realised within a single political term, ambitious interventions can yield almost immediate effects. Modelling shows that within a decade, given sufficient policy ambition, it is possible to achieve significant reductions in infant mortality, nutritional deficiencies, emergency hospital admissions and the number of looked-after children.^71^ Free school meals have been shown to be an effective policy for protecting the most vulnerable children, yet expansion of the scheme for all Universal Credit receiving families was only implemented in September 2026, leaving children vulnerable to another winter of detrimental health effects.^72^ Meanwhile, an expansion to all school children would only have cost £2.5 billion and provided a 1.71 cost–benefit ratio over the next 20 years.^73^ More radical changes to the welfare system come at a larger expense, but also wider benefits. The most ambitious of these may be to legally guarantee all essentials, such as food and heating, as part of Universal Credit, coming at a cost of approximately £22 billion annually, but virtually eliminating destitution and lifting 1.7 million people, of whom 600 000 children, out of poverty completely.^74^

Using evidence-based arguments like the ones presented in this paper, summarised in table 1, to refute common narratives based on misconceptions is a crucial first step in advocacy, building a foundation for lasting change. Given the complex and worsening nature of child poverty in the UK, a concerted and sustained policy drive is required to achieve meaningful progress in both the immediate and longer term. To ensure such a drive is both effective and sustainable, future research should focus on the longitudinal impacts of specific policy interventions underlying any significant reform. For government policy to be truly evidence-based, the conclusions drawn in current and future academic literature must be actively placed into the public sphere. This review equips public health professionals with the evidence needed to support the translation of academic consensus into the media and public discourse, an essential route to achieving the political leverage required for sustained structural reform.

**Table 1:** Summary of evidence supporting structural interventions to tackle child poverty and inequity

Policy domain	Strategic principle	Key supporting evidence
Social investment as a driver of growth	Social investment acts as a critical driver for economic productivity and growth	Examples of other high-income Western societies have shown that maintaining a robust social welfare state does not limit economic competitiveness, but rather incentivises valuable entrepreneurship, outperforming the UK in the process.
		Child poverty will cost the UK government £40 billion annually by 2027, while effective and immediate interventions, such as an extension of free school meals or removal of further benefit caps are available at a fraction of the cost.
Family well-being and structural inequalities	Structural environment and equitable wealth distribution dictates family outcomes and choices	Families living in poverty suffer from structural inequalities that limit their ability to make health-promoting choices for their children; for example, meeting healthy eating guidelines requires 70% of disposable income for the poorest families.
		65% of children living in poverty have at least one working parent, while there is no evidence that welfare funds are widely misused on health-detrimental goods. Nearly half of UK parents skip meals to feed their children.
		Inequalities in educational attainment and opportunity are present at every level of the system, making it harder for children growing up in poverty to break from their disadvantaged background on merit alone.
Structural capacity in housing and public services	Domestic investment determines availability of social services and housing rather than external demographic factors	The housing crisis is primarily driven by domestic policy and market failures, including unregulated private sector rents increasing twice as fast as wages and a deficit of 1.4 million social homes since 1980.
		Blaming immigration for housing shortages ignores that most migrants are ineligible for social housing on arrival, while asylum seekers cannot access council housing until formally granted refugee status.
		Rather than draining public services, most estimates show that migrants contribute more to the economy and taxes than they consume. The entire Home Office spend, which includes immigration and asylum processes, represents just 0.5% of government spending, a fraction of the annual cost of child poverty.

## Supplementary material

10.1136/bmjpo-2026-004714 online supplemental file 1
