# Supplementary material for: Overcoming arguments against tackling child poverty and inequalities: marshalling the evidence
Source: BMJ Paediatr Open. 2026 Nov 2;10(1):e004714. doi: 10.1136/bmjpo-2026-004714 (PMC13735355; doi:10.1136/bmjpo-2026-004714)
Supplement: Supplementary file 1 [file bmjpo-2026-004714.reviewer_comments.pdf]

PEER REVIEW HISTORY

BMJ Paediatrics Open publishes all reviews undertaken for accepted manuscripts. Reviewers are asked to complete a checklist review form and are provided with free text boxes to elaborate on their assessment. These free text comments are reproduced below.

ARTICLE DETAILS

**TITLE (PROVISIONAL)**	Overcoming arguments against tackling child poverty and inequalities: Marshalling the evidence
**AUTHORS**	Limburg, Maximilian<br>Cronin, Mary<br>Pickett, Kate<br>Taylor-Robinson, David

VERSION 1 – REVIEW

**REVIEWER NAME**	Lenton, Simon
**REVIEWER AFFILIATION**	
**REVIEWER CONFLICT OF INTEREST**	No Conflict of Interest
**AI DISCLOSURE**	No, I have not used AI
**DATE REVIEW RETURNED**	22-Mar-2026

**GENERAL COMMENTS**	This is a well-written paper which addresses the three dominant narratives raised in the 2024 deliberative workshop. It could be published in its current form.<br>I was slightly surprised that macroeconomic/fiscal priorities did not reach the top three, although partly covered by ‘social investment’. A short discussion of a living wage versus a minimum wage might be helpful since benefits paid to full-time employees, are essentially a subsidy to businesses paying the minimum wage.<br>Figure 6.2. Deep child poverty rates (after housing costs are deducted) over time in the IFS Report R335 Child poverty: trends and policy options is possibly a better diagram than the figure 1 chosen by the authors.<br>https://ifs.org.uk/sites/default/files/2024-10/Child-poverty-trends-and-policy-options_1.pdf<br>Inclusion of Philip Allston’s report Special Rapporteur on Extreme Poverty and Human Rights, Poverty is a political choice.<br>https://www.ohchr.org/en/statements-and-speeches/2018/11/statement-visit-united-kingdom-professor-philip-alston-united?LangID=E&NewsID=23881 echoes Nelson Mandela’s quotation in Long Walk to Freedom, that “poverty is man-made, meaning that humans can eradicate it” and could be referred to.<br>Figure 14 Change in child income poverty rates, 2012–2014 to 2019–2021 in The UNICEF report card 18 child poverty in the midst of wealth. https://www.unicef.org/innocenti/media/3296/file/UNICEF-Innocenti-Report-Card-18-Child-Poverty-Amidst-Wealth-2023.pdf might be worth including as it demonstrates the UK as being an outlier within developed nations.<br>Minor points<br>• the keywords in the table and the text to not match.<br>• The detrimental impacts of poverty on children’s physical and mental<br>• Pg 3 line 54 health, as well as their long-term life chances - include

	learning here<br>• clarify “social investments”, ‘welfare reform’ and ‘macro-economic policy’ for readers not familiar with the terms

**REVIEWER NAME**	Price, Anna
**REVIEWER AFFILIATION**	Royal Children's Hospital Melbourne, Centre for Community Child Health
**REVIEWER CONFLICT OF INTEREST**	I know the authors through an international network (INRICH) that I co-lead. However, we are not collaborators.
**AI DISCLOSURE**	I was a reviewer for the original submission (ADC) and asked co-pilot to check the author revisions, as they aren't tracked or explicitly described in this next submission. I also asked it to help me write my response.
**DATE REVIEW RETURNED**	08-Apr-2026

**GENERAL COMMENTS**	I reviewed an earlier version of this manuscript for its previous submission, and I am grateful to the authors for the care they have taken to respond thoughtfully to reviewer feedback and to clarify the intent of the paper. The reframing of the deliberative workshop and media review as scoping and validation exercises for a narrative review is helpful.<br>My main remaining concern relates to transparency rather than substance. The paper centres on three dominant narratives underpinning resistance to action on child poverty, but the manuscript still provides limited information to help the reader assess how these narratives were identified in relation to other well-described frames in UK media and policy discourse, or how confident one should be that alternative dominant narratives were not overlooked. Without some justification or acknowledgement of these limitations, it remains difficult to judge whether these themes reflect dominance in the media, salience to advocates, or necessary author judgement.<br>This does not detract from the strength or relevance of the evidence marshalled, but addressing this point explicitly would further strengthen the clarity and credibility of the review.

**REVIEWER NAME**	Waterston, Tony
**REVIEWER AFFILIATION**	Newcastle University, Institute of Health and Society
**REVIEWER CONFLICT OF INTEREST**	No Conflict of Interest
**AI DISCLOSURE**	No, I have not used AI
**DATE REVIEW RETURNED**	08-Apr-2026

**GENERAL COMMENTS**	I commend the authors for this thorough and authoritative review which makes a convincing case for government to put in place policies to reverse the trend to increasing child poverty in the UK. I have seen the authors' responses to referee comments and they have made appropriate changes. I have only two small suggestions to make. First, the authors have described policies in Scandinavian countries to reduce child poverty but could also mention Scotland, where child poverty has been reduced by reducing housing costs and through tax and benefit changes. https://ifs.org.uk/news/scotlands-child-poverty-rate-lower-rest-uk-ambitious-2030-targets-set-be-missed Second, the authors do not make clear how a paper in an academic journal can influence

	government policy. There could be mention of the need for government policy to be evidence based and for the conclusions of this paper to be placed in the public sphere of discussion through publication in the media - a route that is no doubt well known to these authors. I concur that such influence is hard to achieve but always necessary.

VERSION 1 – AUTHOR RESPONSE

- **Author response:**
- **We would like to thank the reviewers for their constructive and positive feedback to the submitted review. We have attempted to incorporate their comments into the existing paper making minor changes to the content throughout. Below, we have addressed the reviewer comments point-by-point.**

Reviewer: 1
Dr. Simon Lenton
Comments to the Author
This is a well-written paper which addresses the three dominant narratives raised in the 2024 deliberative workshop. It could be published in its current form.
I was slightly surprised that macroeconomic/fiscal priorities did not reach the top three, although partly covered by 'social investment'. A short discussion of a living wage versus a minimum wage might be helpful since benefits paid to full-time employees, are essentially a subsidy to businesses paying the minimum wage.

- **Author response:**
- **This has been added to the section on social investment**

Figure 6.2. Deep child poverty rates (after housing costs are deducted) over time in the IFS Report R335 Child poverty: trends and policy options is possibly a better diagram than the figure 1 chosen by the authors.
https://ifs.org.uk/sites/default/files/2024-10/Child-poverty-trends-and-policy-options_1.pdf

- **Author response:**
- **While the proposed figure presents deep child poverty well, it detracts from the purpose of the existing Figure 1, which is to showcase children as the population group suffering from the highest levels of poverty. As such, we chose to keep the initial figure 1.**

Inclusion of Philip Allston's report Special Rapporteur on Extreme Poverty and Human Rights, Poverty is a political choice. https://www.ohchr.org/en/statements-and-speeches/2018/11/statement-visit-united-kingdom-professor-philip-alston-united?LangID=E&NewsID=23881 echoes Nelson Mandela's quotation in Long Walk to Freedom, that "poverty is man-made, meaning that humans can eradicate it" and could be referred to.

- **Author response:**
- **Many thanks for the author for this poignant statement by the former UN Special Rapporteur. This has been added into the manuscript.**

Figure 14 Change in child income poverty rates, 2012–2014 to 2019–2021 in The UNICEF report card 18 child poverty in the midst of wealth. https://www.unicef.org/innocenti/media/3296/file/UNICEF-

Innocenti-Report-Card-18-Child-Poverty-Amidst-Wealth-2023.pdf
might be worth including as it demonstrates the UK as being an outlier within developed nations.

- ➔ **<u>Author response:</u>**
- ➔ **Adding this figure to clarify the previously only verbal description of the Innocenti Report card was a constructive suggestion and has been added.**

Minor points
• the keywords in the table and the text to not match.
• The detrimental impacts of poverty on children's physical and mental
• Pg 3 line 54 health, as well as their long-term life chances - include learning here
• clarify "social investments", 'welfare reform' and 'macro-economic policy' for readers not familiar with the terms

- ➔ **<u>Author response</u>:**
- ➔ **The keywords suggested by the authors are not able to be selected in the submission portal. If this can't be changed during the editorial process, we will keep the ones selected in the portal.**
- ➔ **Minor suggested changes have been made throughout the manuscript.**

Reviewer: 2
Dr. Anna Price, Royal Children's Hospital Melbourne, Murdoch Childrens Research Institute
Comments to the Author
I reviewed an earlier version of this manuscript for its previous submission, and I am grateful to the authors for the care they have taken to respond thoughtfully to reviewer feedback and to clarify the intent of the paper. The reframing of the deliberative workshop and media review as scoping and validation exercises for a narrative review is helpful.
My main remaining concern relates to transparency rather than substance. The paper centres on three dominant narratives underpinning resistance to action on child poverty, but the manuscript still provides limited information to help the reader assess how these narratives were identified in relation to other well-described frames in UK media and policy discourse, or how confident one should be that alternative dominant narratives were not overlooked. Without some justification or acknowledgement of these limitations, it remains difficult to judge whether these themes reflect dominance in the media, salience to advocates, or necessary author judgement.
This does not detract from the strength or relevance of the evidence marshalled, but addressing this point explicitly would further strengthen the clarity and credibility of the review.

- ➔ **<u>Author response:</u>**
- ➔ **An addition has been made to the Introduction (/Methodology) to acknowledge the limitation of allowing a degree of author and participant judgement in the review when selecting the three key narratives.**

Reviewer: 3
Dr. Tony Waterston, Newcastle University
Comments to the Author
I commend the authors for this thorough and authoritative review which makes a convincing case for government to put in place policies to reverse the trend to increasing child poverty in the UK. I have seen the authors' responses to referee comments and they have made appropriate changes. I have only two small suggestions to make. First, the authors have described policies in Scandinavian

countries to reduce child poverty but could also mention Scotland, where child poverty has been reduced by reducing housing costs and through tax and benefit changes. https://ifs.org.uk/news/scotlands-child-poverty-rate-lower-rest-uk-ambitious-2030-targets-set-be-missed Second, the authors do not make clear how a paper in an academic journal can influence government policy. There could be mention of the need for government policy to be evidence based and for the conclusions of this paper to be placed in the public sphere of discussion through publication in the media - a route that is no doubt well known to these authors. I concur that such influence is hard to achieve but always necessary.

- **Author response:**
- **Both suggestions have been added to the manuscript and significantly contribute to the impact of the paper. Many thanks to the reviewer.**

VERSION 2 – REVIEW

**REVIEWER NAME**	Lenton, Simon
**REVIEWER AFFILIATION**	
**REVIEWER CONFLICT OF INTEREST**	No Conflict of Interest
**AI DISCLOSURE**	No, I have not used AI
**DATE REVIEW RETURNED**	19-May-2026

**GENERAL COMMENTS**	excellent - publish!

**REVIEWER NAME**	Price, Anna
**REVIEWER AFFILIATION**	Royal Children's Hospital Melbourne, Centre for Community Child Health
**REVIEWER CONFLICT OF INTEREST**	No Conflict of Interest
**AI DISCLOSURE**	I used AI to check the reviewers' comments against the tracked changes because they didn't specify the new/revised content in the responses document.
**DATE REVIEW RETURNED**	29-May-2026

**GENERAL COMMENTS**	Please see above: This comment relates to the comments I made in two earlier reviews of this paper. I appreciate that the authors have provided a little more information on how they selected the narratives. However, I think a small amount of additional detail on the workshop itself would strengthen the paper. Specifically, the number and type of participants, and the general approach used to elicit or agree on themes. I recognise that this paper is not intended as a formal qualitative study, but a brief description of these elements will help readers understand how the perspectives were generated and whether they reflect a shared or broader view.

**REVIEWER NAME**	Waterston, Tony
**REVIEWER AFFILIATION**	Newcastle University, Institute of Health and Society
**REVIEWER CONFLICT OF INTEREST**	No Conflict of Interest
**AI DISCLOSURE**	No, I have not used AI
**DATE REVIEW RETURNED**	01-Jun-2026

GENERAL COMMENTS	I am happy that the authors have addressed the issues raised by referees and that the paper is now acceptable for publication, congratulations to all the authors for their thoroughness throughout.

VERSION 2 – AUTHOR RESPONSE

- **Author response:**
- **We would like to thank the reviewers for their continued input to ensure the high standards we endeavour to with our submission. Below, we have addressed the reviewer comments point-by-point and hope to have addressed any remaining concerns in the process.**

Reviewer: 1
Dr. Simon Lenton
Comments to the Author
excellent - publish!

Reviewer: 2
Dr. Anna Price, Royal Children's Hospital Melbourne, Murdoch Childrens Research Institute
Comments to the Author
Please see above: This comment relates to the comments I made in two earlier reviews of this paper. I appreciate that the authors have provided a little more information on how they selected the narratives. However, I think a small amount of additional detail on the workshop itself would strengthen the paper. Specifically, the number and type of participants, and the general approach used to elicit or agree on themes. I recognise that this paper is not intended as a formal qualitative study, but a brief description of these elements will help readers understand how the perspectives were generated and whether they reflect a shared or broader view.

- **Author response:**
- The paper has been modified to expand on the methodology of the workshop in the introduction section to address the Reviewer 2 comments. We hope this achieves the desired clarity on the process underlying the evidence synthesis underpinning this 'Review' paper.

Reviewer: 3
Dr. Tony Waterston, Newcastle University
Comments to the Author
I am happy that the authors have addressed the issues raised by referees and that the paper is now acceptable for publication, congratulations to all the authors for their thoroughness throughout.
