# Supplementary material for: Overcoming arguments against tackling child poverty and inequalities: marshalling the evidence
Source: BMJ Paediatr Open. 2026 Nov 2;10(1):e004714. doi: 10.1136/bmjpo-2026-004714 (PMC13735355; doi:10.1136/bmjpo-2026-004714)
Supplement: Supplementary file 2 [file bmjpo-10-1-s001.docx]

| Social investment as a driver of growth | Family well-being and structural inequalities | Structural capacity in housing and public services |
| --- | --- | --- |
| Keir Starmer: “There will be no magic money tree economics with us” – Daily Mirror, 2022 ^A1^ | George Osborne: “Dependency cannot be a lifestyle choice” – Telegraph, 2011 ^B1^ | Spiralling immigration, both legal and illegal, is now clearly exacerbating the UK’s crippling homes shortage – The Telegraph, 2025 ^C1^ |
| Britain still acts as if it has money to burn – The Times, 2025 ^A2^ | David Cameron: “Labour infantilised millions with welfare state” – The Telegraph, 2015 ^B2^ | “It’s not xenophobic to say that mass and rapid migration is unsustainable in terms of housing supply, public services and community relations” – The Guardian, 2023 ^C2^ |
| “Promised to scrap the two-child limit […] “giveaways funded by promises of highly uncertain spending cuts – isn’t this all a little Lizz Truss?” – The Times, 2025 ^A3^ | “Scrapping the two-child limit – an obsession of the progressive left – would hand around £3500 per child annually to large, low-income families” – The Times, 2025 ^B3^ | “Mass migration figures […] are deeply concerning and have serious implications for the housing crisis, crime rates and quality of life across Britain” – GB News, 2025 ^C3^ |
| Boris Johnson: “You don’t promote growth, you don’t make people richer, by spending yet more money on welfare.” – Daily Mail, 2024 ^A4^ | “Ultimately, child poverty is a problem for the parents” – Financial Times, 2025 ^B4^ | “Brits on low pay – and those out of work – are forced to compete with millions of people from abroad for jobs” – Guardian, 2016 ^C4^ |
| “There is one forecast I feel confident in making: That the people who are contributing least to economic growth will be the ones reaping the rewards” – The Sun, 2024 ^A/B5^ | | “He’ll go for growth – even if it means extra housing pressure and lower salaries” – The Telegraph, 2023 ^C5^ |
| “Like the Soviet Union in its last decades, Britain would have become the land which economic development forgot.” – The Sun, 2024 ^A6^ | The welfare system is “promoting destructive behaviour” by encouraging poorer families to have more children and denying them the incentive to get a job – The Telegraph, 2012 ^B6^ | “When immigration is too high, when the pace of change is too fast, it’s impossible to build a cohesive society. It’s difficult for schools and hospitals and core infrastructure like housing and transport to cope” – The Spectator, 2015 ^C6^ |
| Common themes identified through media review: | | |
| Pro-growth economic policies are essential to lifting people out of poverty, e.g. lower taxes, reduced government spending, free trade | Parents bear responsibility for the wealth of their families and children. The government is not responsible for personal choices | Immigration takes priority over essential services and depletes the welfare state, diverting important resources from children |
| Streamline welfare with work requirements, while economic policies increase labour force participation, productivity and economic mobility in order to reduce overall dependency on government support | Receiving and maintaining welfare needs to be made more difficult to avoid ‘benefit lifestyle’. Giving equal opportunity to children should give them the tools to lift themselves out of poverty if they are willing. | Urgently reduce net migration and illegal migration, removing social protections of migrants if necessary |

**Table S1:** Results of search on media discourse, References to articles in Table S2

**Table S2:** References for media search

| Nr. | Citation |
| --- | --- |
| A1 | Mirror Online. Keir Starmer vows 'no magic money tree economics' in dig at Tory leadership hopefuls [online]. 2022. <https://www.mirror.co.uk/news/politics/keir-starmer-vows-no-magic-27563746.amp> (accessed 29 April 2025) |
| A2 | The Sunday Times. Britain still acts as if it has money to burn [online]. [Britain still acts as if it has money to burn](https://www.thetimes.com/comment/columnists/article/voters-must-accept-harsh-realities-of-growth-jd83rwl62?msockid=3aa022b8e30b6c1425b53702e2716db5). 2025. <https://www.thetimes.com/comment/columnists/article/voters-must-accept-harsh-realities-of-growth-jd83rwl62?msockid=3aa022b8e30b6c1425b53702e2716db5> (accessed 2 May 2025) |
| A3 | The Sunday Times. What Nigel Farage gets right about economic policy [online]. 2025. <https://www.thetimes.com/business-money/economics/article/what-nigel-farage-gets-right-about-economic-policy-2xbj9tmcd?msockid=3aa022b8e30b6c1425b53702e2716db5> (accessed 18 June 2025) |
| A4 | Daily Mail. BORIS JOHNSON enters the election fray and declares: There's no doubt that Keir Starmer would be the most dangerous and Left-wing prime minister since the 1970s [online]. 2024. <https://www.dailymail.co.uk/debate/article-13456697/Boris-Johnson-Keir-Starmer-dangerous-left-wing-1970s.html> (accessed 29 April 2025) |
| A6 | The Sun. Labour promised 'growth, growth, growth'… if they mean it, cut public sector and boost private sector [online]. 2024. <https://www.thesun.co.uk/news/32509294/labour-growth-cut-public-sector-ross-clark/> (accessed 29 April 2025) |
| B1 | The Telegraph. Dependency cannot be a lifestyle choice [online]. 2011. <https://www.telegraph.co.uk/news/politics/8331776/Dependency-cannot-be-a-lifestyle-choice.html> (accessed 2 May 2025) |
| B2 | The Telegraph. Labour 'infantilised' millions with welfare state, David Cameron says [online]. 2015. <https://www.telegraph.co.uk/news/politics/david-cameron/11417964/Labour-infantalised-millions-with-welfare-state-David-Cameron-says.html> (accessed 29 April 2025) |
| B3 | The Sunday Times. What Nigel Farage gets right about economic policy [online]. 2025. <https://www.thetimes.com/business-money/economics/article/what-nigel-farage-gets-right-about-economic-policy-2xbj9tmcd?msockid=3aa022b8e30b6c1425b53702e2716db5> (accessed 18 June 2025) |
| B4 | Financial Times. Ultimately, child poverty is an issue for the parents [online]. 2025. <https://www.ft.com/content/7e5750ef-ca20-4874-9d3a-fa3a73a46df2> (accessed 29 Aug 2025) |
| A/B5 | The Sun. Labour promised 'growth, growth, growth'… if they mean it, cut public sector and boost private sector [online]. 2024. <https://www.thesun.co.uk/news/32509294/labour-growth-cut-public-sector-ross-clark/> (accessed 2 May 2025) |
| B6 | The Telegraph. Benefits encourage problem families, says Iain Duncan Smith [online]. 2012. <https://www.telegraph.co.uk/news/politics/conservative/9631820/Benefits-encourage-problem-families-says-Iain-Duncan-Smith.html> (accessed 2 May 2025) |
| C1 | GB News. Migrant crisis: New data reveals shocking immigration truth...and it'll be a 'disaster' for Britain [online]. 2025. <https://www.gbnews.com/membership/migrant-crisis-newdata-shocking-immigration-truth-disaster-britain> (accessed 29 Aug 2025) |
| C2 | The Spectator. Theresa May lambasts her own record on immigration. Why? [online]. 2015. <https://www.spectator.co.uk/article/theresa-may-lambasts-her-own-record-on-immigration-why/> (accessed 2 May 2025) |
| C3 | The Guardian. Are EU migrants really taking British jobs and pushing down wages? [online]. 2016. <https://www.theguardian.com/politics/2016/may/20/reality-check-are-eu-migrants-really-taking-british-jobs> (accessed 2 May 2025) |
| C4 | The Telegraph. I’ve changed my mind about why Britain has a chronic housing shortage [online]. 2025. <https://www.telegraph.co.uk/business/2025/07/27/immigration-fuelling-uk-chronic-housing-shortage/?msockid=053e3bfdec6765b5324e2da7ed7464a1> (accessed 29 Aug 2025) |
| C5 | The Guardian. Suella Braverman to rebuff cabinet calls for easing of visa rules [online]. 2023. <https://www.theguardian.com/politics/2023/may/14/suella-braverman-to-rebuff-cabinet-calls-for-easing-of-visa-rules> (accessed 2 May 2025) |
| C6 | The Telegraph. The Tories have a dirty secret: they don’t want to reduce immigration [online]. 2023. <https://www.telegraph.co.uk/news/2023/05/25/the-tories-dont-want-to-reduce-immigration/> (accessed 2 May 2025) |
